# Emerging scrub typhus infection in the northern region of Sri Lanka

**DOI:** 10.1186/1756-0500-7-719

**Published:** 2014-10-14

**Authors:** Jebananthy Anandaselvam Pradeepan, Natkunam Ketheesan, Kalamathy Murugananthan

**Affiliations:** Department of Medicine, Faculty of Medicine, University of Jaffna, Jaffna, Sri Lanka; Australian Institute of Tropical Health and Medicine, James Cook University, Townsville City, Australia; Department of Pathology, Faculty of Medicine, University of Jaffna, Jaffna, Sri Lanka

**Keywords:** Rickettsioses, Scrub typhus (ST), ELISA IgM and IgG, Eschar, Sri Lanka

## Abstract

**Background:**

There is an increasing trend in rickettsioses or typhus fevers in the island of Sri Lanka. The seroepidemiological mapping previously published did not include the northern region of the island. This study was conducted to demonstrate the presence of scrub typhus (ST) and to characterise the clinical presentation of ST in this region.

**Findings:**

Serum samples from patients (n = 64) with clinical symptoms suspected of typhus fever following exclusion of other common febrile illnesses commonly seen in the northern region of Sri Lanka were selected and screened for ST using specific IgM and IgG ELISA (ImBios, USA). ST was confirmed by serology in 54 patients, with typical eschar being found in 49 of cases positive for ST. Fever was the sole presenting complaint of these patients with the duration of febrile illness varying from 2–14 days. Of these patients 44.4% had regional lymphadenopathy, 18.5% hepatomegaly, 12.9% pneumonitis and 9.3% splenomegaly. None of the patients had a rash.

**Conclusions:**

This study confirms the presence of high numbers of patients with ST in northern Sri Lanka. It was found that 84.4% of the patients presenting with clinical features of rickettsioses (54 of the 64) were seropositive for ST with a significant majority having a typical eschar. This data provided will enable clinicians to be vigilant of ST in this region and provide appropriate therapy and also facilitate planning for preventive measures aimed at reducing the burden of ST.

## Findings

### Background

The incidences of rickettsial diseases are increasing internationally. Since 1937 there have been several reports of rickettsiosis from the island of Sri Lanka [1–5]. In 2011, Liyanapathirana and Thevanesam published the first comprehensive island wide sero-epidemiological map of rickettsial diseases with data from eight of the nine provinces of the country [6]. They found that spotted fever group (SFG) was more prevalent in the central hilly regions covering two provinces of the country whereas scrub typhus (ST) was more common in the dry zone which are the peripheral provinces. However, this study did not include the Northern Province of the island (third largest province in the country covering 14% of the total land mass) due to the civilian conflict which prevailed at that time. The Jaffna Teaching Hospital, in the Northern Province of Sri Lanka is the tertiary hospital for this region serving 2.9% of the total population of Sri Lanka. The third highest notification rate of rickettsiosis in the country is reported from the Jaffna Teaching Hospital [7]. The current study was carried out among patients presenting with typhus fever like symptoms to the Jaffna Teaching Hospital. The objective of this study was two fold: confirmation by serological tests that the patients clinically suggestive of having typhus fever had in fact ST and to describe the spectrum of clinical presentation in these patients.

### Methods

Ethical approval for this study was obtained from the Ethical Review Committee of the Faculty of Medicine, University of Jaffna. This study included all patients above the age of 12 admitted to the Medical Wards of the Jaffna Teaching Hospital, between March 2012 and March 2013 with illness clinically suggestive of ST. Parental consent was obtained for those participants under the age of 18. Following informed written consent, patient details, clinical history and epidemiological data were obtained and entered using a pre tested pro forma. The Surveillance Case Definitions for Notifiable Diseases in Sri Lanka [8] criteria were used to identify the patients for this study. These include acute febrile illness associated with eschar, headache, macular papular skin rash, conjunctival injection, lymphadenopathy, profuse sweating and cough. Defervescence within 48 hours following tetracycline therapy was also considered to be strongly suggestive of rickettsial infection. Patients with other febrile illnesses with obvious causes including respiratory, urinary tract and skin infections were excluded from this study. Patients admitted with febrile illnesses common to the region such as typhoid fever and dengue which were confirmed by rapid diagnostic tests were also excluded from this study. A total of 64 patients were selected initially for this study. All cases negative for ST ELISA were excluded from further analysis. Samples that were positive for either ST IgM or positive for both ST IgM and ST IgG were considered as having acute ST.

Serum samples from freshly collected venous blood were stored at -40°C. All serological tests were performed on stored samples and IgM and IgG tests for ST were performed using ELISA kits (ImBios, USA) according to manufacturer’s instructions. The Cut-off value for the assay was calculated by determining the mean optical density plus three times the standard deviation of normal human serum. Samples with spectrophotometric readings more than the cut off value were considered positive for ST. All the tests were performed in duplicates.

### Results

Of the 64 initial patient samples, 54 were positive for ST. The mean age of those positive for ST was 34.6 ± 17.6.Of the patients with ST, 72.2% (n = 39) were females and 64.8% (n = 35) were from rural areas. Fever was the only presenting complaint. The fever duration varied from 2–14 days (mean 7.3 ± 2.6). Eschar was found in 49 of the 54 patients positive for ST (90.7%). The eschar was distributed around the trunk, inguinal region, genitalia, axilla and limbs (39.2%, 31.4%, 11.7%, 11.7% and 5.8% respectively). None of the ST seropositive patients had a rash (Table 1). Of these patients 44.4% had regional lymphadenopathy, 18.5% hepatomegaly, 12.9% pneumonitis and 9.3% splenomegaly. The only patient who suffered encephalitic features was found to have co-infection of both dengue and ST. The white blood cell count was within the normal range in 64% of the patients with leucopoenia in 17.2% and leucocytosis in 9.3%. A significant proportion of patients (46.9%) had platelet counts of less than < 1.5 x 10^9^/L.

**Table 1:** **Clinical features of patients with scrub typhus**

	Northern Sri Lanka (this study)	Western Sri Lanka [9]	Thailand [10]
**Confirmed cases (n)**	54	20	30
**Mean fever duration**	7.5	11	9
**Escher (%)**	90.7	25	68
**Lymphadenopathy (%)**	44.4	60	93
**Hepatomegaly (%)**	18.5	95	73
**Splenomegaly (%)**	9.3	35	23
**Headache (%)**	11.1	20	NS
**Pneumonitis (%)**	12.9	45	NS
**Rash (%)**	0	0	30
**Diarrhoea (%)**	0	5	27

NS not specified.

### Discussion

The number of notifications of rickettsioses in Sri Lanka has increased 13 fold over a period of 20 years [11]. This increase could be due to actual increase in the incidence of rickettsioses, increased awareness and/or improved notification. Previous studies have clearly shown that ST is predominant in the dry zone of Sri Lanka [6, 12]. Since the Northern Province is in the dry zone, it was considered that ST would be the predominant cause of rickettsioses in this region. Therefore serum samples from 64 patients identified clinically as possibly having ST were included in this retrospective study. Due to resource limitations these serum samples were tested using only IgM and IgG ELISA specific for ST. Of the samples tested 54 were found to be positive for ST.

The presence of an eschar could be pathognomonic for ST infection and enables clinical diagnosis. But, if the bite occurred in hidden areas of the body the eschar may not be obvious. An eschar is formed by the bite of chigger mite that inoculates *Orientia tsutsugamushi*, the causative agent of ST. All the patients in this study who had an eschar were positive for ST by serology. However, not all patients with positive ST serology had an eschar. Some studies [13] describe that only a small percentage of patients with ST have eschars. However, most studies from Sri Lanka, Thailand, China, Korea and Japan have reported the presence of eschars in a high proportion of patients with ST (68% to 93%) [10, 14–16]. Although the presence of eschar could be specific, the painless nature of this dermatological manifestation and its location in the body may not attract the attention of patients. It may therefore pose a diagnostic challenge for clinicians. In this study, it was found that the eschar was mainly confined to the trunk, inguinal, genital and axially areas of the patients. Similar pattern distribution was observed in studies carried out in India [9] which also revealed a significant difference in the distribution of eschars between males and females. While the location of eschars is common on the chest and abdomen among females, the axilla, groin and genitalia areas were more common in males. It is good clinical practice in ST endemic region to thoroughly examine patients who may be suspected of having ST for the presence of eschar, in particular in covered areas of the body. None of the patients in the current study had a rash. This is consistent with publications from other parts of Sri Lanka [13]. However, rash in patients with ST has been reported to be 30% in studies carried out in Thailand [10], 90% in China [15] and 93% in Japan [16]. In a proportion of patients with ST, eschar and or rash may not be present. In such cases when ST is suspected in the absence of eschar or rash, it is important to exclude other febrile illnesses common to a particular region before considering treatment with doxycycline. Regular monitoring of temperature is recommended to determine the effectiveness of doxycycline therapy.

Complications of ST vary depending on the organ systems involved. Complications associated with hypovolemia could lead to vasculitis and thrombosis. Rare complications also include uraemia, kidney failure, myocarditis, pneumonia, respiratory failure, and the involvement of the central nervous system. In this study a low platelet count was observed in 46.9% of the study population. Low platelet counts may trigger suspicion of some other acute febrile illnesses prevalent in the region such as dengue. In this study, the only patient who presented with meningo-encephalitis and was found to be seropositive for both dengue fever and ST. This patient’s condition improved with treatment with doxycycline.

Of the patients included in this study, the majority were from rural and agricultural areas with females making up of more than two third of the patient cohort. Females in rural areas are more likely to be exposed to the larval mite bite possibly due to their involvement in agricultural activities in areas with vegetation. A similar pattern and distribution of ST was noted in a study recently conducted in Southern India [17]. Environmental factors including seasonality, vegetation and population density also contribute to the spread of ST [18]. Rodents act as reservoir host for the mite larvae. More than half of the patients confirmed the presence of rats in their dwellings. Enclosed areas are more conducive for rodents to breed and may contribute to the incidents of ST. The observations that ST is predominant in the northern region of Sri Lanka would enable personnel working in preventive health to create awareness and increase educational activities that would lead to the control of mites and rodents in addition to current efforts in eliminating ticks on companion animals to prevent other rickettsial infections.

Currently there is no vaccine available for preventing rickettsial infections. Antibiotics are not recommended for prophylaxis to travellers to endemic areas. However, travellers should be instructed to minimize exposure to infectious arthropods and use of proper insect or tick repellents. Wearing protective clothing and self-examination after visits to vector-infested areas are recommended. Such precautions are important, in particular for people with underlying conditions that may increase susceptibility to rickettsial infections.

### Conclusion

There is an increasing trend in rickettsial diseases in Sri Lanka. This study confirms that a majority of patients presenting with clinical features of rickettsial diseases in the northern region of Sri Lanka may have ST.

## Authors’ information

JA Pradeepan is a Lecturer in Medicine at the Medical Faculty, University of Jaffna, Sri Lanka and a General Physician attached to the Jaffna Teaching Hospital and has an interest in Infectious Diseases. N Ketheesan is the Professor of Infection and Immunity at the Australian Institute of Tropical Health and Medicine, James Cook University, Australia and has an interest in host-pathogen interactions in infectious diseases relevant to the tropics. K Murugananthan is a Senior Lecturer in Microbiology at the University of Jaffna, Sri Lanka and has an interest in Rickettsial diseases and Medical Virology.
